# Pathogenicity of rice yellow mottle virus and screening of rice accessions from the Central African Republic

**DOI:** 10.1186/s12985-017-0912-4

**Published:** 2018-01-08

**Authors:** Regis Dimitri Sokpe Longue, Valentin Stanislas Edgar Traore, Innocent Zinga, Maxwell Darko Asante, Zakaria Bouda, James Bouma Neya, Nicolas Barro, Oumar Traore

**Affiliations:** 1 Laboratory of Biological and Agronomic Sciences for Development (LaSBAD), Life Science Department, University of Bangui, BP 908 Bangui, Central African Republic; 2 Institute of Environment and Agricultural Research (INERA), Ouagadougou, 01 BP 476 Burkina Faso; 3 Department of Biochemistry and Microbiology, University of Ouagadougou I-Professor Joseph Ki-Zerbo, Ouagadougou, Burkina Faso; 4 Concil for Scientific and Industrial Research –Crop Research Institute, CSIR-CRI, P.O. Box 3785, Kumasi, Ghana

**Keywords:** *RYMV*, Resistance breaking isolates, Pathogenicity, rice accessions

## Abstract

**Background:**

Rice yellow mottle virus (RYMV) of the genus *Sobemovirus* is the most important viral pathogen of rice causing more damage to rice crop in Sub Saharan Africa. The aim of this study was to conduct pathogenic characterization of RYMV isolates from the Central African Republic (CAR) and to screen commonly cultivated rice accessions in the country for resistance/tolerance to the virus.

**Methods:**

The pathogenicity of RYMV isolates was studied by mechanical inoculation with comparison to differential rice lines highly resistant to RYMV available at the Institute of Environment and Agricultural Research (INERA) in Burkina Faso.

To screen commonly cultivated rice accessions in CAR, characterized RYMV isolates from the country were used as inoculum sources. Resistant breaking (RB) isolates were used to prepare RB-inoculum, whereas non-resistant breaking isolates (nRB) were used for nRB-inoculum.

**Results:**

Overall 102 isolates used in this study, 29.4% were able to overcome the high resistance genes in the rice cultivars Gigante and Tog7291. All isolates were distributed within three distinct pathogenic profiles. The first profile constituted of 6.9% of the isolates was able to break down the resistance in rice cultivar Gigante only. The second pathogenic profile made of 19.6% of isolates was able to infect Tog7291 only. The third profile, 2.9% of isolates overcame simultaneously resistance genes in both rice cultivars Gigante and Tog7291. Out of isolates able to break down the resistance gene in cultivar Gigante, a single isolate was found to be non-infectious to the susceptible control IR64.

Data from screening showed that all accessions were susceptible to RYMV, although IRAT213 was found to be partially resistant to both nRB-inoculum and RB-inoculum.

**Conclusion:**

The present study can be considered as the first in the Central African Republic, it gives a caution on the high risk of RYMV damage to rice production in the country. Beside, skills of pathogenic profiles of RYMV isolates will contribute to better disease management.

**Electronic supplementary material:**

The online version of this article (10.1186/s12985-017-0912-4) contains supplementary material, which is available to authorized users.

## Background

Pests and diseases represent about 30% yield loss in staple food crops, worldwide [1]. Currently, the most widely used strategy for disease control is genetic resistance, through the use of resistant varieties against pathogens. However, the ability of pathogens to overcome resistant genes in crops compromised the durability of these resistances.

Rice yield losses payable to Rice yellow mottle virus (RYMV) were estimated between 20 and 100% [2–4] with important socioeconomic effects for farmers. It has reported that rice varieties Gigante and Bekarosaka species (*Oryza sativa*) as well as *O. glaberrima* species (Tog5681, Tog5672, Tog5674 and Tog7291) were found to be highly resistant to RYMV [5–9]. Highly resistance varieties are characterized by a lack of symptoms expression and undetectable virus content into inoculated plant tissues using ELISA test. Resistance gene *RYMV1* with four independent resistance alleles was identified in both *O. sativa* (allele *rymv1–2*) and *O. glaberrima* species (alleles *rymv1–3*, alleles *rymv1–4* and alleles *rymv1–5*). In addition to *RYMV1*, *RYMV2* and *RYMV3* resistance genes have been described in accessions Tog7291 and Tog5307, respectively [6, 7, 10]. Genetic diversity analysis of RYMV revealed six main strains spread throughout sub-Saharan African rice cultivation regions moving from East to West [11, 12]. In the Central African Republic, despite the recent emergence of RYMV in the country, isolates were also found to be genetically diversified [13]. Indeed, in the most recent study, we reported that isolates from the Central African Republic belong to two sister phylogenetic groups closely related to the S1ca strain, a strain found in eastern West Africa, Cameroon and in Chad, neighboring countries of the CAR [13]. Genetic diversity occurring among RYMV populations seems to present a big challenge for breeding rice for durable resistance to plant virus.

Several studies on the pathogenicity of RYMV were conducted mainly in Burkina Faso. Nearly 40% of RYMV isolates were able to overcome high resistance with different pathways [14, 15]. A different figures of RYMV pathogenicity were found depending on isolates origins, but not necessarily on genetic diversity of isolates [16]. Isolates from East Africa have more ability to overcome resistance of *Oryza sativa* specie such as Gigante, while isolates from West and Central Africa have a high pathogenic diversity. So, it was showed that RYMV strains from west and central Africa have capacity to break down resistance of *Oryza sativa* and *O. glaberrima* species  [7, 14, 17]. In addition, regarding the adaptation of RYMV to host species, the ability to break down resistance was associated to polymorphism in central domain of the VPg, a viral protein covalently linked to the viral genome. So, the T/E polymorphism at VPg codon 49, significantly associated to resistance break down  [16, 17, 18]. In this study, we firstly characterized RYMV isolates from the Central African Republic for their pathogenicity using differential rice varieties to the virus. Secondly, we screened rice varieties from the Central African Republic for resistance using the characterized RYMV isolates.

## Methods

### Plant material

Concerning pathogenicity studies, resistant rice varieties from Institute of Environment and Agricultural Research (INERA) were used including susceptible rice variety IR64 as controls (Table 1). Seven cultivated rice varieties collected from the Institute of Agronomic in Central African Republic (ICRA) were involved in the screening. Out of these rice varieties six (IRAT213, ITA, NL24, NL60, TCS10 and Roc5) were introduced in different international rice intitute and one was a local variety (Ndouroudamba). Rice cv. Gigante and Tog7291 were used as resistant control along with rice cv. Azucena and IR64 used as partially resistant and susceptible varieties, respectively.

**Table 1:** Reference cultivars used in the pathogenicity assessment of RYMV isolates of the Central African Republic

Variéties	Species	Origin	Resistance level^a^	Resistance gene^b^	Reference
IR64	*Oryza sativa* L*.*	INERA	S		
Gigante	*O. sativa* L*.*	INERA	HR	*rymv1–2*	[5, 9]
Tog5672	*O. glaberrima* Steud.	INERA	HR	*rymv1–4/RYMV2*	[5, 6, 9]
Tog5674	*O. glaberrima* Steud.	INERA	HR	*rymv1–5*	[5, 6, 9]
Tog5681	*O. glaberrima* Steud.	INERA	HR	*rymv1–3*	[5, 6, 9]
Tog7291	*O. glaberrima* Steud.	INERA	HR	*RYMV2*	[5, 6, 9]

^a^S (susceptible control), HR (highly resistant control)

^b^*RYMV1* (allele *rymv1–2* to *rymv1–*5) and *RYMV2* are genes conferring high resistance in rice cultivars

### RYMV isolates

Among 2012 and 2015, RYMV isolates were collected in several localities in the south of the CAR. A set of 102 of these isolates were used in this study to assess pathogenicity using resistant rice varieties (Additional file 1: Supplementary Table 1).

### Plant inoculation

For all experiments, virus isolates were firstly propagated in susceptible rice variety IR64. Infected leaf samples from cv. IR64 were then ground into 0.01 M phosphate buffer, pH 7.0 at the ratio 1/10 (*w*/*v*) using sterile mortars and pestles. Carborundum (600 mesh) was added to the extracts, which were subsequently rubbed into leaves of 2-week-old seedlings. Leaves were collected from infected plants 14 days post inoculation (DPI) and used as inoculum source for experiments.

For the pathogenicity experiment, 6 plants (14 days old seedling) of each of the 6 differential varieties were inoculated using every single isolate. All experiments were done in insect-proof conditions to avoid contamination.

For the screening test, two sources of inoculum (nRB-inoculum and RB-inoculum) were used to screen rice varieties. nRB-inoculum was prepared using 8 nRB-isolates in a mixture at equal ratio of infected leaves, while RB-inoculum was made of 2 RB-Gigante isolates or 2 RB-Tog7291 isolates independently as describe by Traore et al. [19]. Thus, two similar experimental layouts were established based on the two inoculum sources. Rice seedlings of 21 days old were inoculated. nRB-inoculum and RB-inoculum were used separately to inoculate 6 plants for each variety, including controls (IR64, Azucena, Gigante and Tog7291).

### Evaluation of resistance-breaking isolates

The pathogenic characterization of RYMV isolates was made by monitoring symptom appearance and severity for 45 days post inoculation (DPI). Disease severity was assessed using standard evaluation scale [20] noted from 1 (no symptoms) to 9 (severe symptoms). At the end of experiments, symptomatic plant leaves were collected individually while asymptomatic leaves were pooled per variety and assayed for virus presence using serological test. Direct double antibody sandwich enzyme-linked immunosorbent assay (DAS-ELISA) was performed to detect the presence of RYMV into leaf extracts as made by Longue et al. [21]. Leaf extracts from 21 DPI plants were assessed at the ratio of 1:10 (*w*/*v*) to estimate virus titers in both symptomatic and asymptomatic plants.

### Statistical analysis

XLSTAT software 2016 (www.xlstat.com) was used for the statistical analyses. Fisher F test was used to compare the variance of the delay of symptoms expression and virus content of leaf samples. Whereas proportions of isolates able to break down high resistance cultivars were compared using a parametric test for the comparison of k proportions. Student t test was used to compare means of virus content of highly resistant cultivars inoculated with nRB-inoculum and RB-inoculum. For *p-*value ≤0.05, differences were considered to be statistically significant.

## Results

### Symptoms expression

Over all tested isolates, the susceptible rice variety IR64 (control) expressed symptoms homogeneously on all plants (100%) between 6 to 10 DPI. On the highly resistant cultivars, 26% of isolates induced visible yellow mottle symptoms on two rice resistant cultivars Gigante and Tog7291. Symptoms appeared in time interval from 22 to 42 DOI depending to rice cultivars. Analysis of variance of the number of days for symptoms appearance indicated a significant cultivar effect (F = 35.91, dd1 = 30, 28; *P* < 0.001). Symptom severities were higher in IR64 than in Gigante and Tog7291, scores (7 to 9 versus 3 to 5). In addition, the rate of infected plants was low, ranged from 1/6 to 2/6 in Gigante and from 2/6 to 4/6 in Tog7291. For all isolates that induced symptoms in resistant cultivars, three profiles could be distinguished. The first concern 7 isolates (27%) which induced symptoms in Gigante only. In the second profile, 16 isolates (61.5%) infected symptomatically Tog7291 only. The last profile consisted to 3 isolates (11.5%) that induced symptoms in both Gigante and Tog7291. It appears clearly that proportion of isolates that induced symptom in Tog7291 was up to two times higher than those infected Gigante. Comparison between proportions of isolates of first and second profile using z test indicated significant difference (z = 2. 88; *P* < 0.01).

### Asymptomatic detected plants

**Fig. 1:**
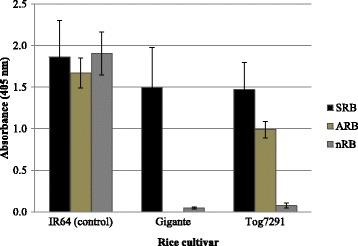
Virus content of highly resistant rice cultivars Gigante and Tog7291. The legend indicated resistance breaking (RB) isolates with symptoms induced (SRB) or no symptoms induced (ARB). nRB indicates non-resistance breaking isolates. The error bars represent the standard deviation of the means

Asymptomatic resistance-breaking isolates (ARB) were detected using direct double antibody sandwich enzyme-linked immunosorbent assay (DAS-ELISA). The test was done in three repetitions per sample. Results indicated that virus isolates were detected in highly resistant cv. Tog7291 only. Thus, 4 isolates infected Tog7291 without induce visible symptoms. The absorbance at 405 nm reflecting viral titer of asymptomatic plants ranged in average from 0.75 to 1.15. In the same way, virus contents of plants with symptomatic infections were estimated. The virus contents were higher in symptomatic infections than in asymptomatic one with absorbance averages of 1.86, 1.50 and 1.47 for cv. IR64 (susceptible control), Gigante and Tog7291, respectively (Fig. 1). Analyze of variance of viral titers in symptomatic and asymptomatic infections in Tog7291 indicated significant difference (F = 5.87; ddl = 10, *P* < 0.05). In contrast, virus content of symptomatic infections in Gigante and in Tog7291 showed no significant different (F = 2,16; ddl = 10, 21; *P* = 0,24). These results suggested that symptoms development could depend on level of virus infection in plants from cv. Tog7291.

**Fig. 2:**
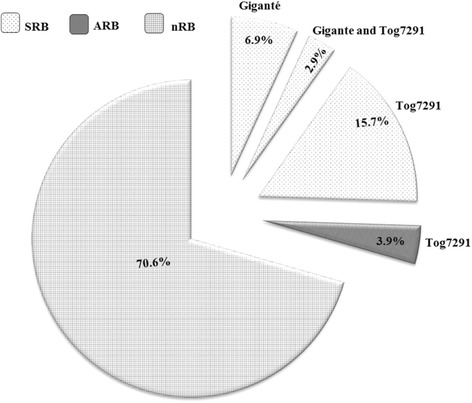
Frequency of isolates that overcame highly resistant cultivars Gigante and Tog7291. RB indicates resistance breaking isolates, with symptomatic (SRB) or asymptomatic infections (ARB) while nRB indicates non-resistance breaking isolates, or isolates that did not induce any infection

Overall, depending on SRB and ARB, the proportion of isolates that overcome resistant rice cultivars Gigante and Tog7291 was 29.4% as summarized in the Fig. 2. Resistance-breaking isolates can be divided in three groups: (i) isolates that infected Gigante alone (6.9%); (ii) isolates which overcome Tog7291 (19.6%) and (iii) those infected both Gigante and Tog7291 (2.9%). The proportion of isolates able to break down resistance of Tog7291 was about three time upper than those overcome Gigante. Consequently, percentage of non-resistance-breaking isolates (nRB) was 70.6%.

Out of 102 isolates tested, no virus has infected varieties Tog5681, Tog5672 and Tog5674, highly resistant to RYMV. The absence of virus detection by ELISA test and symptom expression indicated that those isolates were not able to break down the high resistance of these rice varieties.

### Back inoculation to confirm RB

**Fig. 3:**
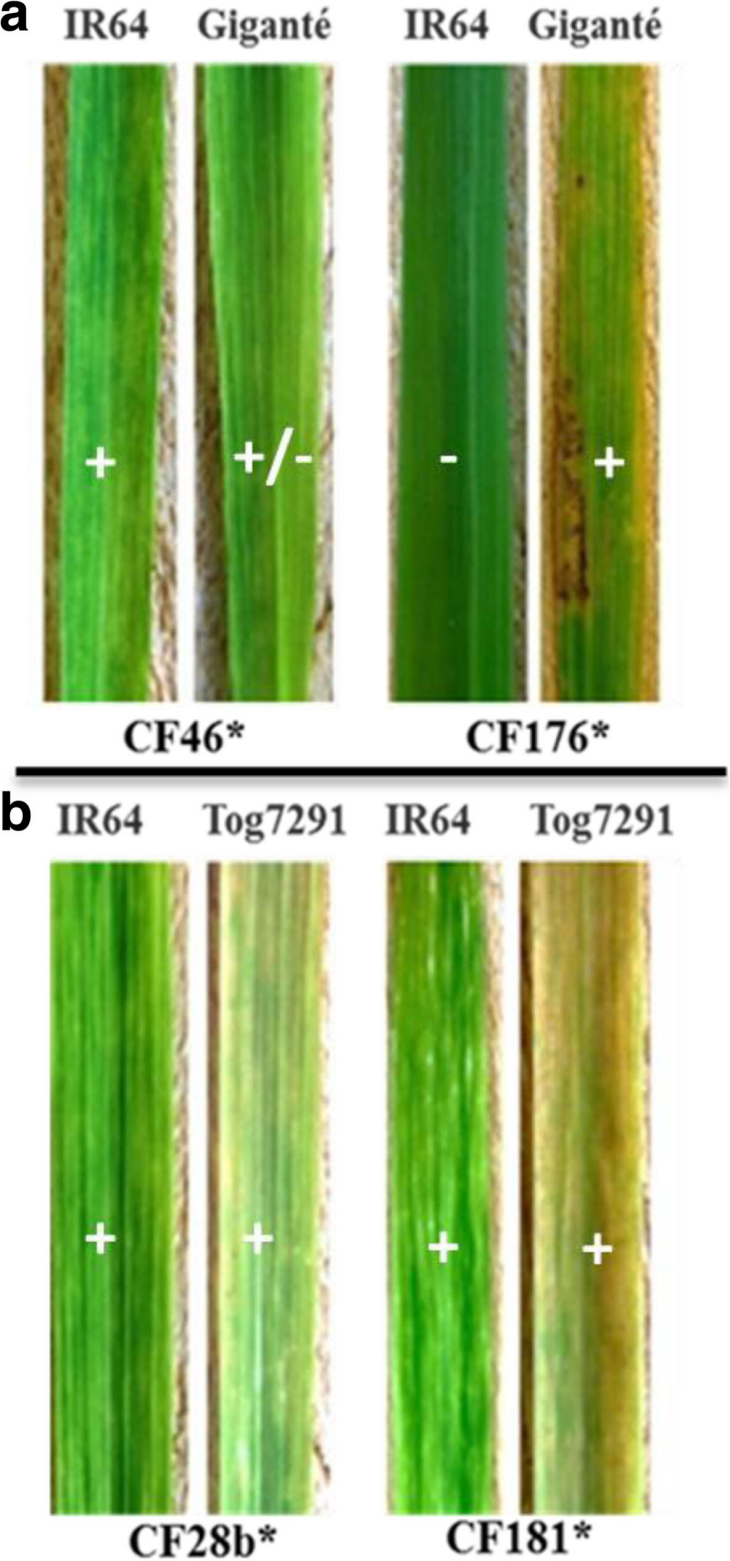
Symptoms expressed by resistance breaking isolates in Gigante (**a**) and Tog7291 (**b**). Rice cultivars are above the corresponding leaves. * indicates virus isolates (virulent variant) that broke down resistance

RYMV isolates that overcome highly resistant cultivars Gigante or Tog7291 were used separately to confirm the resistance-breaking. Infected Gigante or Tog7291 plants were used as inoculum sources for back-inoculation test. All RB isolates induced symptoms between 8 and 12 DPI with incidences ranged from 75 to 100% in Gigante and Tog7291 except CF176* isolate (RB-Gigante). However, IR64 inoculated by RB isolates expressed symptoms on 100% of plants 6 to 8 DPI except CF46 isolate. Indeed, both RB variants CF176* and CF46* presented contrary reactions in cultivars Gigante and IR64. Isolate CF176* inoculated to cultivar Gigante induced symptoms at 10 DPI on over 90% of plants, while CF46* which infected plants at 17 DPI with incidence less than 50%. Conversely and surprisingly, no visible symptoms were observed on cv. IR64 (Fig. 3) and undetectable level of virus in ELISA test. This indicates that CF176* variant that evolved to infect Gigante was associated to a fitness cost leading to the loss of infectivity in susceptible variety IR64.

### Response of rice cultivars to RB or nRB-isolates

All rice accessions were found to be susceptible to RYMV isolates from nRB-inoculum and RB-inoculum except IRAT213. Symptoms were appeared from 8 to 16 DPI in average including cv. IR64 and Azucena, respectively susceptible and partial resistance controls (Table 2). Analyze of variance for the period of symptoms development showed no significant difference between inoculum sources (F = 1.17; ddl = 5, *P* = 0.86). Nevertheless, some differences were found in cv. IRAT213 which reacted differently with nRB-inoculum or RB-inoculum. Based on symptom appearance, IRAT213 reaction with nRB-inoculum showed the same level of resistance than Azucena (13 DPI) whereas with RB-inoculum, IRAT213 showed a higher resistance than Azucena (16 vs 11 DPI). This suggests that IRAT213 reacted as a partial resistant cultivar.

**Table 2:** Number of days for symptom appearance of rice accessions at 21 DPI

	Symptoms expression (DPI)			
Accessions	nRB-Inoculum	susceptibility	RB-Inoculum	susceptibility
IR64 (control)	8	S	8	S
Azucena (control)	13	PR	11	S
Gigante (control)	AS	HR	11	S
Tog7291 (control)	AS	HR	13	S
IRAT 213	13	S	16	PR
ITA	9	S	10	S
Ndouroudamba	10	S	11	S
NL24	11	S	10	S
NL60	9	S	10	S
TCS10	8	S	8	S
Roc5	8	S	8	S

All control cultivars are susceptible, partial resistant and highly resistant to nRB-Inoculum and RB-Inoculum

(AS) indicates no symptoms observed, HR highly resistant and PR partial resistant

### Virus contents of accessions

**Fig. 4:**
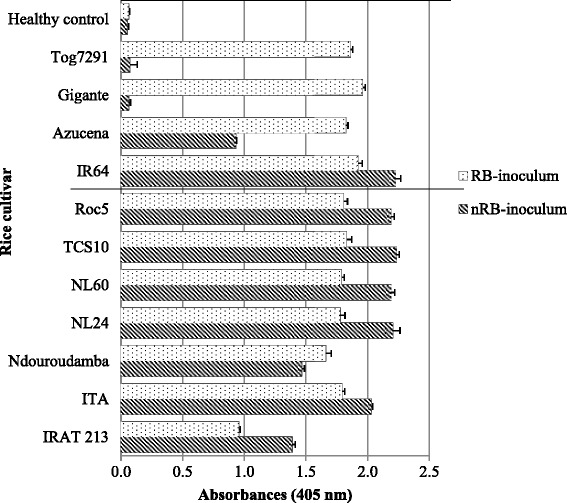
Virus contents of rice accessions challenged with nRB isolates or RB isolates. The error bars represent the standard deviation of the means

As summarized in the Fig. 4, the virus titer in the tested cultivars inoculated with nRB-inoculum was similar for all tested rice varieties except IRAT213 and Ndouroudamba which are intermediate between the susceptible ones and Azucena. For RB-inoculum, only IRAT213 was lower (and similar as nRB-inoculum on Azucena). Compared to Azucena and Tog7291 with RB-inoculum, IRAT213 showed symptoms later and lower virus titer. Student t test to compare the means of virus titers between plants inoculated with nRB-inoculum and RB-inoculum showed no significant difference (*t* = 1,63; ddl = 12, *P* = 0,13). However, virus contents in the cv. IRAT213 inoculated with nRB-inoculum were higher than those of plants inoculated with RB-inoculum.

Globally, highest virus titers were found in rice cultivars ITA, NL24, NL60, TCS10 and Roc5 while the lowest were found in IRAT213 and the local cv. Ndouroudamba inoculated with both nRB-inoculum and RB-inoculum. Despite difference in the number of day for symptoms development, there is no inoculum effect on plants development in cv. IRAT213, as well as, in Azucena (unpublished data), indicating tolerance aspect to RYMV isolates.

## Discussion

This is the first time that the RB ability of isolates from CAR was assessed. Previously, isolates from Cameroon and Chad, neighboring countries, were assessed in Gigante and Tog5681 [14]. In comparison with their results, we noticed that isolates from CAR showed lower ability to overcome Gigante (7% vs 30%) and could not infect Tog5681 (0% vs 9%). In a recent study, Tog7291 was reported to be overcome by 7/8 isolates from Niger, Nigeria and Togo [16]. The high frequency of RB isolates in cultivar Tog7291 indicates easy adaptation of isolates from Central African Republic to this cultivar. Nevertheless, it was shown that isolates that broke down resistance in Gigante were frequently found in Central African region including Cameroon and Chad, countries neighboring the Central African Republic [14]. None of the tested isolates infected cultivar Tog5681 contrary to those belonging to Sudano-savannah region of Cameroon and Chad.

Yet isolates of the South of Central African Republic were phylogenetically a sister group of S1ca (S1 strain from Central Africa region) strain that closely related to S1wa (S1 strain from West Africa) and all originate from a same common ancestor [11–13]. Thus, it is likely that isolates of the Central African Republic inherit this ability to overcome high resistances from the common ancestor. Incapability of isolates from Central African Republic of overcoming the high resistance in Tog5681 distinguishes them to isolates from Sudano-savannah region of Cameroon and Chad. This feature may be have resulted from genetic distance between isolates from West Africa (Niger and Nigeria) closely related to those of Cameroon and Chad [13].

The resistance break down was associated with contrasted fitness cost. RB isolate CF176* (virulent) showed the loss of infectivity in the susceptible cultivar IR64 in agreement with results reported previously [17, 22, 23]. By contrast, RB isolate CF46* have lowest fitness cost in the resistant cultivar Gigante but infect normally control cultivar IR64 as demonstrated by Jenner et al. [24] with *Turnip mosaic virus* RB isolates.

The role of polymorphism was demonstrated at the position 49 in the central domain of the viral protein-link genome (VPg) in ability to break down high resistance [16, 18, 25]. Indeed, isolates with a glutamic acid (E49) were more able to overcome Gigante than those with a threonine (T49) which infects Tog5681 and Tog7291. It would be interesting to continue this study by looking for the molecular basis associated to the RB high resistant cultivars Gigante or Tog7291.

In the second part of this study, we evaluated the behavior of rice accessions grown in the Central African Republic against RYMV isolates (nRB-inoculum and RB-inoculum). The results showed homogeneity of reaction in all rice accessions with both inoculums. This indicates overall susceptibility of rice accessions in the Central African Republic to RYMV isolates, limited to those used. Recently, in similar studies, Traore et al. [19] reported significant inoculum effects in accessions used, while our results show no difference between both nRB and RB isolates. This difference could be due, on the one hand, to differences in pathogenicity between the RYMV isolates and, on the other hand, to different accessions used in these two studies. As partial resistance to RYMV characterized by a delay in symptom expression and a lower virus accumulation [26], thus the rice variety IRAT213 may be considered as partially resistant to RB isolates. In response to the current epidemic, the use of this rice cultivar would prevent significant damages. However, this study is very limited regarding the number of accessions screened. Then, rice accessions should be collected in the country in order to better understand their resistance or susceptibility level to RYMV isolates.

## Conclusion

The pathogenicity pathways of RYMV isolates from the south of the Central African Republic are diverse and could help to better manage of genetic control of rice yellow mottle disease. The screening of some rice accessions permitted to understand the risk of devastation for the rice production in the country.

## Additional file

Additional file 1: Table S1. List and origin of isolates used in this study. (DOCX 33 kb)

## Abbreviations

ARB: Asymptomatic resistance breaking isolates
DAS-ELISA: Double antibody sandwich enzyme-linked immunosorbent assay
DPI: Day post inoculation
ICRA: Central African Republic institute of agronomic research
INERA: Institute of Environment and Agricultural Research
nRB: Non-resistance breaking isolates
RB: Resistance breaking isolates
RYMV: Rice yellow mottle virus
SRB: Symptomatic resistance breaking isolates
VPg: Viral protein-link genome
