# Supplementary material for: Pathogenicity of rice yellow mottle virus and screening of rice accessions from the Central African Republic
Source: Virol J. 2018 Jan 8;15:6. doi: 10.1186/s12985-017-0912-4 (PMC5759187; doi:10.1186/s12985-017-0912-4)
Supplement: Additional file 1: Table S1. — List and origin of isolates used in this study. (DOCX 33 kb) [file 12985_2017_912_MOESM1_ESM.docx]

Additional file 1: Table S1. List and origin of isolates used in this study.

| **Isolates** | **Country** | **Locatlty** | **Strain^a^** | **Year** | **Pathogenic profile^b^** |
| --- | --- | --- | --- | --- | --- |
| CF5 | Central African Republic | M'poko | S1ac | 2013 | *nRB* |
| CF8 | Central African Republic | M'poko | S1ac | 2013 | *nRB* |
| CF16 | Central African Republic | M'poko | S1ac | 2013 | *RB Tog7291* |
| CF17 | Central African Republic | M'poko | S1ac | 2013 | *nRB* |
| CF20 | Central African Republic | M'poko | S1ac | 2013 | *nRB* |
| CF22 | Central African Republic | M'poko | S1ac | 2013 | *nRB* |
| CF23 | Central African Republic | M'poko | S1ac | 2013 | *RB Togg7291* |
| CF26 | Central African Republic | M'poko | S1ac | 2013 | *RB Gigante* |
| CF28b | Central African Republic | M'poko | S1ac | 2013 | *RB Tog7291* |
| CF19 | Central African Republic | M'poko | S1ac | 2013 | *RB Tog7291* |
| CF77 | Central African Republic | Ngola | S1ac | 2014 | *RB Gigante* |
| CF78 | Central African Republic | Ngola | S1ac | 2014 | *RB Tog7291* |
| CF79 | Central African Republic | Ngola | S1ac | 2014 | *nRB* |
| CF131 | Central African Republic | Ngola | S1ac | 2014 | *RB Tog7291* |
| CF81 | Central African Republic | Ngola | S1ac | 2014 | *RB Tog7291* |
| CF82 | Central African Republic | Ngola | S1ac | 2014 | *nRB* |
| CF92 | Central African Republic | Ngola | S1ac | 2014 | *nRB* |
| CF83 | Central African Republic | Ngola | S1ac | 2014 | *RB Gigante* |
| CF85 | Central African Republic | Ngola | S1ac | 2014 | *nRB* |
| CF87 | Central African Republic | Ngola | S1ac | 2014 | *nRB* |
| CF163 | Central African Republic | Ngola | S1ac | 2014 | *nRB* |
| CF90 | Central African Republic | Ngola | S1ac | 2014 | *nRB* |
| CF91 | Central African Republic | Ngola | S1ac | 2014 | *nRB* |
| CF93 | Central African Republic | Ngola | S1ac | 2014 | *RB Gigante* |
| CF94 | Central African Republic | Ngola | S1ac | 2014 | *RB Tog7291* |
| CF86 | Central African Republic | Ngola | S1ac | 2014 | *nRB* |
| CF96 | Central African Republic | Ngola | S1ac | 2014 | *RB Tog7291* |
| CF132 | Central African Republic | Ngola | S1ac | 2014 | *nRB* |
| CF133 | Central African Republic | Ngola | S1ac | 2014 | *RB Gig/Tog* |
| CF134 | Central African Republic | Ngola | S1ac | 2014 | *RB Gig/Tog* |
| CF135 | Central African Republic | Ngola | S1ac | 2014 | *RB Tog7291* |
| CF145 | Central African Republic | Ngola | S1ac | 2014 | *RB Tog7291* |
| CF137 | Central African Republic | Ngola | S1ac | 2014 | *nRB* |
| CF146 | Central African Republic | Ngola | S1ac | 2014 | *nRB* |
| CF150 | Central African Republic | Ngola | S1ac | 2014 | *RB Tog7291* |
| CF149 | Central African Republic | Ngola | S1ac | 2014 | *nRB* |
| CF140 | Central African Republic | Ngola | S1ac | 2014 | *RB Tog7291* |
| CF141 | Central African Republic | Ngola | S1ac | 2014 | *nRB* |
| CF148 | Central African Republic | Ngola | S1ac | 2014 | *nRB* |
| CF147 | Central African Republic | Ngola | S1ac | 2014 | *nRB* |
| CF139 | Central African Republic | Ngola | S1ac | 2014 | *RB Tog7291* |
| CF151 | Central African Republic | Ngola | S1ac | 2014 | *nRB* |
| CF155 | Central African Republic | Ngola | S1ac | 2014 | *nRB* |
| CF160 | Central African Republic | Ngola | S1ac | 2014 | *nRB* |
| CF162 | Central African Republic | Ngola | S1ac | 2014 | *nRB* |
| CF166 | Central African Republic | Ngola | S1ac | 2014 | *nRB* |
| CF103 | Central African Republic | Ngola | S1ac | 2014 | *nRB* |
| CF111 | Central African Republic | Ngola | S1ac | 2014 | *nRB* |
| CF115 | Central African Republic | Ngola | S1ac | 2014 | *nRB* |
| CF128 | Central African Republic | Ngola | S1ac | 2014 | *RB Gig/Tog* |
| CF119 | Central African Republic | Ngola | S1ac | 2014 | *nRB* |
| **Supplementary table 1.** Continue | | |  |  |  |
| CF129 | Central African Republic | Ngola | S1ac | 2014 | *RB Tog7291* |
| CF165 | Central African Republic | Ngola | S1ac | 2014 | *nRB* |
| CF126 | Central African Republic | Ngola | S1ac | 2014 | *RB Tog7291* |
| CF138 | Central African Republic | Ngola | S1ac | 2014 | *nRB* |
| CF152 | Central African Republic | Ngola | S1ac | 2014 | *RB Tog7291* |
| CF113 | Central African Republic | Ngola | S1ac | 2014 | *nRB* |
| CF122 | Central African Republic | Ngola | S1ac | 2014 | *nRB* |
| CF110 | Central African Republic | Ngola | S1ac | 2014 | *RB Tog7291* |
| CF153 | Central African Republic | Ngola | S1ac | 2014 | *nRB* |
| CF109 | Central African Republic | Ngola | S1ac | 2014 | *nRB* |
| CF136 | Central African Republic | Ngola | S1ac | 2014 | *nRB* |
| CF127 | Central African Republic | Ngola | S1ac | 2014 | *RB Gigante* |
| CF124 | Central African Republic | Ngola | S1ac | 2014 | *nRB* |
| CF164 | Central African Republic | Ngola | S1ac | 2014 | *RB Tog7291* |
| CF158 | Central African Republic | Ngola | S1ac | 2014 | *nRB* |
| CF63 | Central African Republic | Sabiri | S1ac | 2014 | *nRB* |
| CF64 | Central African Republic | Sabiri | S1ac | 2014 | *nRB* |
| CF181 | Central African Republic | Sabiri | S1ac | 2015 | *RB Tog7291* |
| CF176 | Central African Republic | Sabiri | S1ac | 2015 | *RB Gigante* |
| CF27 | Central African Republic | Sakaï | S1ac | 2014 | *nRB* |
| CF30 | Central African Republic | Sakaï | S1ac | 2014 | *nRB* |
| CF31 | Central African Republic | Sakaï | S1ac | 2014 | *nRB* |
| CF32 | Central African Republic | Sakaï | S1ac | 2014 | *nRB* |
| CF38 | Central African Republic | Sakaï | S1ac | 2014 | *nRB* |
| CF39 | Central African Republic | Sakaï | S1ac | 2014 | *nRB* |
| CF40 | Central African Republic | Sakaï | S1ac | 2014 | *nRB* |
| CF41 | Central African Republic | Sakaï | S1ac | 2014 | *nRB* |
| CF42 | Central African Republic | Sakaï | S1ac | 2014 | *nRB* |
| CF43 | Central African Republic | Sakaï | S1ac | 2014 | *nRB* |
| CF44 | Central African Republic | Sakaï | S1ac | 2014 | *nRB* |
| CF45 | Central African Republic | Sakaï | S1ac | 2014 | *nRB* |
| CF46 | Central African Republic | Sakaï | S1ac | 2014 | *RB Gigante* |
| CF47 | Central African Republic | Sakaï | S1ac | 2014 | *nRB* |
| CF49 | Central African Republic | Sakaï | S1ac | 2014 | *nRB* |
| CF50 | Central African Republic | Sakaï | S1ac | 2014 | *nRB* |
| CF52 | Central African Republic | Sakaï | S1ac | 2014 | *nRB* |
| CF53 | Central African Republic | Sakaï | S1ac | 2014 | *nRB* |
| CF54 | Central African Republic | Sakaï | S1ac | 2014 | *nRB* |
| CF55 | Central African Republic | Sakaï | S1ac | 2014 | *nRB* |
| CF56 | Central African Republic | Sakaï | S1ac | 2014 | *nRB* |
| CF58 | Central African Republic | Sakaï | S1ac | 2014 | *nRB* |
| CF59 | Central African Republic | Sakaï | S1ac | 2014 | *nRB* |
| CF60 | Central African Republic | Sakaï | S1ac | 2014 | *nRB* |
| CF61 | Central African Republic | Sakaï | S1ac | 2014 | *nRB* |
| CF62 | Central African Republic | Sakaï | S1ac | 2014 | *nRB* |
| CF68 | Central African Republic | Sakaï | S1ac | 2014 | *nRB* |
| CF69 | Central African Republic | Sakaï | S1ac | 2014 | *nRB* |
| CF70 | Central African Republic | Sakaï | S1ac | 2014 | *nRB* |
| CF71 | Central African Republic | Sakaï | S1ac | 2014 | *nRB* |
| CF72 | Central African Republic | Sakaï | S1ac | 2014 | *nRB* |
| CF73 | Central African Republic | Sakaï | S1ac | 2014 | *nRB* |

^a^S1ca: RYMV strain found in the central african region; ^b^RB and nRB: Resistance Breaking and non-Resistance Breaking isolates, respectively.
